# Gun Access and Safety Practices among Older Adults

**DOI:** 10.1155/2016/2980416

**Published:** 2016-02-02

**Authors:** Hillary D. Lum, Hanna K. Flaten, Marian E. Betz

**Affiliations:** ^1^VA Eastern Colorado GRECC, Denver, CO 80022, USA; ^2^Division of Geriatric Medicine, University of Colorado School of Medicine, Aurora, CO 80045, USA; ^3^Department of Emergency Medicine, University of Colorado School of Medicine, Aurora, CO 80045, USA

## Abstract

*Background*. Given high rates of gun ownership among older adults, geriatric providers can assess firearm safety practices using a “5 Ls” approach: Locked; Loaded; Little children; feeling Low; and Learned owner. This study describes gun access and the “5 Ls” among US older adults.* Methods*. Data on the “5 Ls” from the Second Injury Control and Risk Survey (ICARIS-2), a national telephone survey conducted by the Centers for Disease Control and Prevention, were analyzed. Weighted variables were used to generate national estimates regarding prevalence of gun ownership and associated gun safety among older adults (≥55 years).* Results*. Of 2939 older adults, 39% (95% CI 37%–42%) reported ≥1 gun stored at home. Among those with guns at home, 21% (95% CI 18–24%) stored guns loaded and unlocked; 9.2% (95% CI 6.6–12%) had ≥1 child in household; 5.1% (95% CI 3.5–6.8%) reported past-year suicidal ideation and 3.6% (95% CI 2.1–5.2%) reported history of a suicide attempt; and 55% (95% CI 51–59%) stated that ≥1 adult had attended firearm safety workshop.* Conclusion*. Some older adults may be at elevated risk of firearm injury because of storage practices, suicidal thoughts, or limited safety training. Future work should assess effective approaches to reduce the risk of gun-related injuries among older adults.

## 1. Introduction

Gun safety among older adults is relevant to medical and public health professionals, given that older adults experience changes in memory, function, and mood that may increase risks of gun-related injuries and death, including suicide [1, 2]. Older adults have high rates of gun ownership [3], potentially placing individuals, families, and home healthcare providers at risk [4]; injury risk rises when guns are stored loaded and unlocked [5]. Older adults have high rates of suicide, and gun access is a risk factor for suicide [6–8]. Healthcare practitioners may play an important role in assessing gun safety and risk of injury in older adults [4, 9–11].

However, currently there are limited data on gun safety among older adults who own guns [12, 13]. Recent work proposed that clinicians ask older adults “Is there a firearm in the home?” and use a “5 Ls” framework to assess safety: (1) Is it Loaded? (2) Is it Locked? (3) Are Little children present? (4) Is the operator feeling Low? (5) Is the operator Learned? [10]. Using the “5 Ls” framework, this study employs the national Second Injury Control and Risk Survey (ICARIS-2) to examine the prevalence and characteristics of older adult gun owners, as well as gun safety related to firearm storage, suicidal thoughts, presence of children, and firearm safety training.

## 2. Methods

### 2.1. Survey Design and Participants

The Centers for Disease Control and Prevention conducted the ICARIS-2 survey between July 2001 and February 2003 [14]. The cross-sectional, random-digit-dial telephone survey included English- and Spanish-speaking adults aged ≥18 years in US households. The response rate was 48% [14]. This analysis includes adults aged ≥55 (*n* = 29390), who accounted for 29% of all respondents. Adults aged 55 years and older were chosen for this analysis because the survey was conducted approximately 10 years ago. Thus, inclusion of individuals 55 years and older reflects a US population cohort that are adults aged 65 and older at the time of this analysis. Analysis was at the level of the individual; survey weighting variables were used to generate national estimates. Weighting was conducted according to the recommended methods as described and recommended for interpretation of survey results [14]. This study was approved by the Colorado Multiple Institutional Review Board.

### 2.2. Variables

Demographic characteristics included age, gender, self-described race, and Hispanic ethnicity. Household characteristics included living alone, presence of children (<18 years), and annual household income. Presence of a firearm was based on the question “Any firearms kept in/around home in the past 12 months?” Participants who did not know (*n* = 5) or declined to answer (*n* = 64) were excluded.

Using the “5 Ls” framework, analysis addressed firearm storage (“Locked” and “Loaded”); presence of children in the home (“Little children”); suicidal thoughts and prior attempts (“feeling Low”); and prior firearm safety training (“Learned operator”). Survey wording (“Were any firearms ever kept loaded and unlocked while stored in or around your home”?) prevented separation of locked and loaded in terms of storage patterns.

### 2.3. Statistical Analysis

All responses were summarized using weighted proportions and 95% Confidence Intervals (CI). Characteristics of respondents with and without firearms at home were compared using Chi Square analyses. Analyses were performed using Stata, version 11.2, with appropriate operations for weighted survey data.

## 3. Results

**Figure 1:**
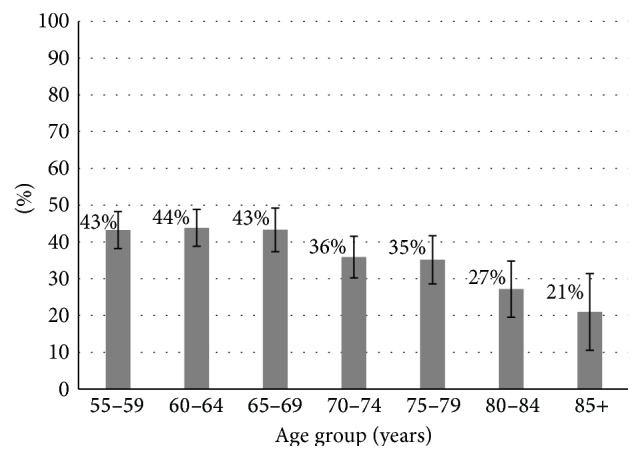
Older adults with ≥1 gun at home, by age group (*N* = 2939). Weighted proportion of older adults with ≥1 firearm in the home, by age group. Error bars represent 95% Confidence Intervals.

The survey respondents were 56% female, and 22% were at least 75 years old at the time of the survey. In older adults aged ≥55 years, 39% (95% CI 37%–42%) reported ≥1 gun stored in or around the home.  Figure 1 shows the percentages of individuals with at least 1 firearm in the home by age group. Of individuals with a gun at home, 65% (95% CI 62%–69%) said ≥1 was a handgun and 69% (95% CI 65%–72%) reported being the owner of the gun, with a higher proportion of men (92%, 95% CI 89–94%) than women (40%, 95% CI 33–46%) reporting being the owner (*p* < 0.001).

Proportions of respondents reporting a gun at home was higher in the younger cohorts (ages 55–64 and 65–74) and among respondents who were white, had less formal education, and had income above the poverty level (Table 1). Firearm presence at home was lower among individuals living alone. The prevalence of households with a gun varied by region, with the highest levels in the Mountain region (55%, 95% CI 46%–65%) and lowest in the Mid-Atlantic region (23%, 95% CI 18%–28%).

**Figure 2:**
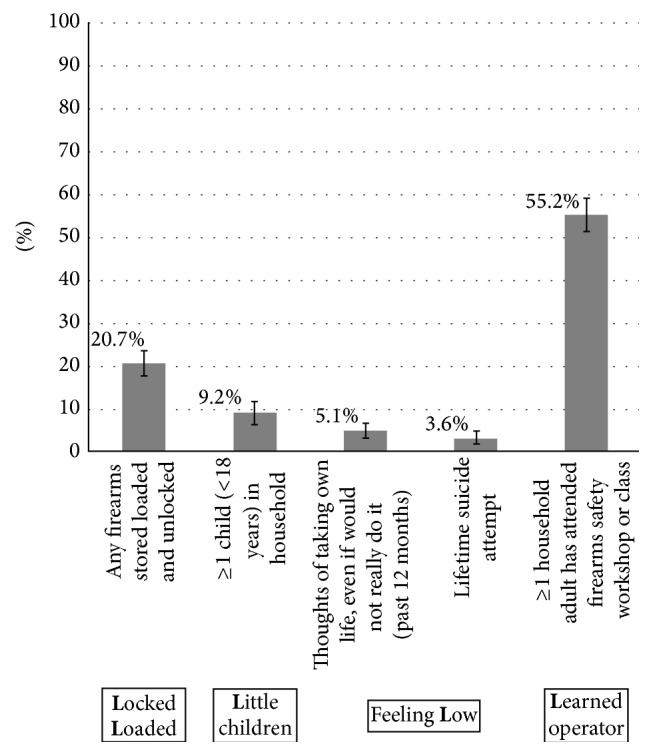
Gun safety practices among older adults with ≥1 gun at home. Weighted prevalence of the 5 Ls framework for gun safety among older adult gun owners (*N* = 987). Error bars represent 95% Confidence Intervals.

Figure 2 shows the prevalence of potential gun safety risks associated with having a gun at home using the “5 Ls” framework [10]. While the prevalences of children in the household, suicidal ideation, and history of suicide attempt were low, one-fifth (21%, 95% CI 18–24%) of gun owners reported having kept the gun loaded and unlocked at some point over the past year. Those with a child in the household were more likely to report safe gun storage (i.e., unloaded and locked; 90%, 95% CI 82–98%; versus 77%, 95% CI 73–80%; *p* < 0.01). Only about half (55%; 95% CI 51–59%) of respondents with a gun at home reported that at least one adult household member had attended a firearm safety workshop or class. There was no association between storing a gun loaded and unlocked and having at least one adult who had attended gun safety training (*p* = 0.8).

## 4. Discussion

In this national survey of US older adults, nearly 40% reported a gun in the home, and approximately 1 out of 5 respondents with a gun at home reported that the gun was stored loaded and unlocked. Given the risks for gun-related injuries among older adults, these findings support the importance of considering firearm safety in older adults. The relevance of healthcare providers asking about gun storage is emphasized by studies demonstrating a high prevalence of loaded firearms in households including individuals with dementia (45%) [15] or children (50%) [16]. In this study, less than 10% with a gun at home reported that a child under age 18 lived in the household and those with children in the home were more likely to report safe gun storage, consistent with other reports [17]. In this era of heightened awareness about firearm-related injuries, healthcare practitioners play an important role in assessing gun safety [4, 18, 19]. Although the ICARIS-2 survey did not ask respondents about cognitive or functional status, future studies, as well as clinical practice, should include detailed assessment of these domains that are also important to the overall safety of older adults and may interact with gun safety practices.

Although the report of suicidal ideation and history of suicide attempt were both low, the importance of assessing risk for self-harm must be emphasized. Rates of firearm suicide rise with age, peaking among those aged 80 to 84 years at an age-adjusted rate of 13.3 per 100,000 [20], and a newly acquired firearm warrants further evaluation due to increased mortality risk [6, 15, 21]. Men account for over 90% of all suicides among those aged 70 years and older [20]. Given the evidence of “lethal means restriction” (i.e., restricting access to guns and other lethal methods of suicide) as a suicide prevention approach [22, 23], healthcare providers should assess firearm access in anyone with suicidal thoughts or behaviors.

There are limitations in interpreting these results. While the overall response rate was only 48%, the weighting variable adjusts for over- or undersampling and nonparticipation and the survey's wide sampling frame further strengthens its generalizability to community-dwelling US adults. Second, the survey relies on self-report of potentially sensitive topics (firearms and suicidal thoughts) without external verification for accuracy. Social desirability may have biased responses to sensitive questions, although this would likely be in a direction that would overestimate gun safety. Temporal changes in gun ownership are also a potential limitation, as current gun safety practices may have changed since the survey was conducted. Still, these data are the most recent national estimates with information about the “5 Ls” framework at the individual level.

## 5. Conclusions

This study provides information about firearm safety practices among US older adults. It emphasizes the potentially elevated risk of firearm injury because of storage practices, suicidal thoughts, or limited firearm safety training. Geriatric practitioners and other health care providers can use the “5 Ls” framework to identify older adults potentially at risk for firearm injury, including those storing guns loaded and unlocked, those with suicidal ideation, and those with limited formal gun safety training. These findings support the need for research to identify effective approaches to assess gun safety and reduce the risk of gun-related injuries among older adults and their household members.

## Figures and Tables

**Table 1:** Characteristics of older adults by presence of ≥1 gun stored in or around the home.

	Gun in the home			No gun in the home			Total		
	Weighted %^a^	95% CI		Weighted %^a^	95% CI		Weighted %^a^	95% CI	
Age group^*∗∗*^									
55–64	47	43	51	39	36	42	42	40	45
65–74	36	33	40	36	33	39	36	34	38
75–84	15	13	18	21	19	23	19	17	21
85+	1.7	0.7	2.6	4.1	3.0	5.2	3.1	2.4	3.9
Female	44	40	48	63	61	66	56	54	58
Race^b^									
White^*∗∗*^	89	86	92	79	77	82	83	81	85
Black^*∗∗*^	6.0	4.1	7.9	12	10	14	9.6	8.2	11
Other	2.7	1.5	3.9	4.5	3.1	5.9	3.8	2.8	4.8
Live alone^*∗∗*^	16	14	18	30	28	32	25	23	26
Income below poverty level^*∗*^	43	39	47	48	45	52	46	44	49
Education^*∗*^									
College or higher	34	30	37	36	33	39	35	33	37
High school	26	23	30	24	21	27	25	23	27
Below high school	40	36	44	40	37	43	40	38	42
Census division (with included states)^*∗∗*^									
New England (CT, MA, ME, NH, RI, VT)	4.7	2.9	6.4	6.7	5.1	8.3	5.9	4.7	7.1
Mid-Atlantic (NJ, NY, PA)	8.9	6.8	11	20	18	22	15	14	17
East North Central (IL, IN, MI, OH, WI)	15	12	17	16	13	18	15	14	17
West North Central (IA, KS, MN, MO, ND, NE, SD)	7.2	5.2	9.3	6.9	5.5	8.4	7.1	5.9	8.3
South Atlantic (DC, DE, FL, GA, MD, NC, WV, SC, VA)	20	17	23	18	16	21	19	17	21
East South Central (AL, KY, MS, TN)	8.6	6.5	11	4.6	3.4	5.8	6.2	5.1	7.3
West South Central (AR, LA, OK, TX)	13	10	15	8.1	6.4	9.7	9.8	8.5	11
Mountain (AZ, CO, ID, MT, NM, NV, UT)	9.6	7.1	12	5.0	3.8	6.3	6.8	5.6	8.1
Pacific (AK, CA, HI, OR, WA)	14	11	16	15	13	17	14	12.6	16.1

*N* = 2,939. CI: Confidence Interval. ^a^Survey data includes weighting variables for generation of national estimates. ^b^Multiple responses allowed per participant; ^*∗*^ *p* ≤ 0.05; ^*∗∗*^ *p* ≤ 0.001 under Pearson chi-square tests; numbers may not add to 100% due to rounding.
